# Neuregulin 1: an intriguing therapeutic target for neurodevelopmental disorders

**DOI:** 10.1038/s41398-020-00868-5

**Published:** 2020-06-16

**Authors:** Liang Shi, Clare M. Bergson

**Affiliations:** 1 Department of Pharmacology and Toxicology, Medical College of Georgia at Augusta University, 1460 Laney Walker Boulevard, Augusta, GA 30912 USA; 2 Present Address: Department of Cell Biology, Emory University School of Medicine, Atlanta, GA USA

**Keywords:** Molecular neuroscience, Psychiatric disorders

## Abstract

Neurodevelopmental psychiatric disorders including schizophrenia (Sz) and attention deficit hyperactivity disorder (ADHD) are chronic mental illnesses, which place costly and painful burdens on patients, their families and society. In recent years, the epidermal growth factor (EGF) family member Neuregulin 1 (NRG1) and one of its receptors, ErbB4, have received considerable attention due to their regulation of inhibitory local neural circuit mechanisms important for information processing, attention, and cognitive flexibility. Here we examine an emerging body of work indicating that either decreasing NRG1–ErbB4 signaling in fast-spiking parvalbumin positive (PV+) interneurons or increasing it in vasoactive intestinal peptide positive (VIP+) interneurons could reactivate cortical plasticity, potentially making it a future target for gene therapy in adults with neurodevelopmental disorders. We propose preclinical studies to explore this model in prefrontal cortex (PFC), but also review the many challenges in pursuing cell type and brain-region-specific therapeutic approaches for the NRG1 system.

## Introduction

**Fig. 1:**
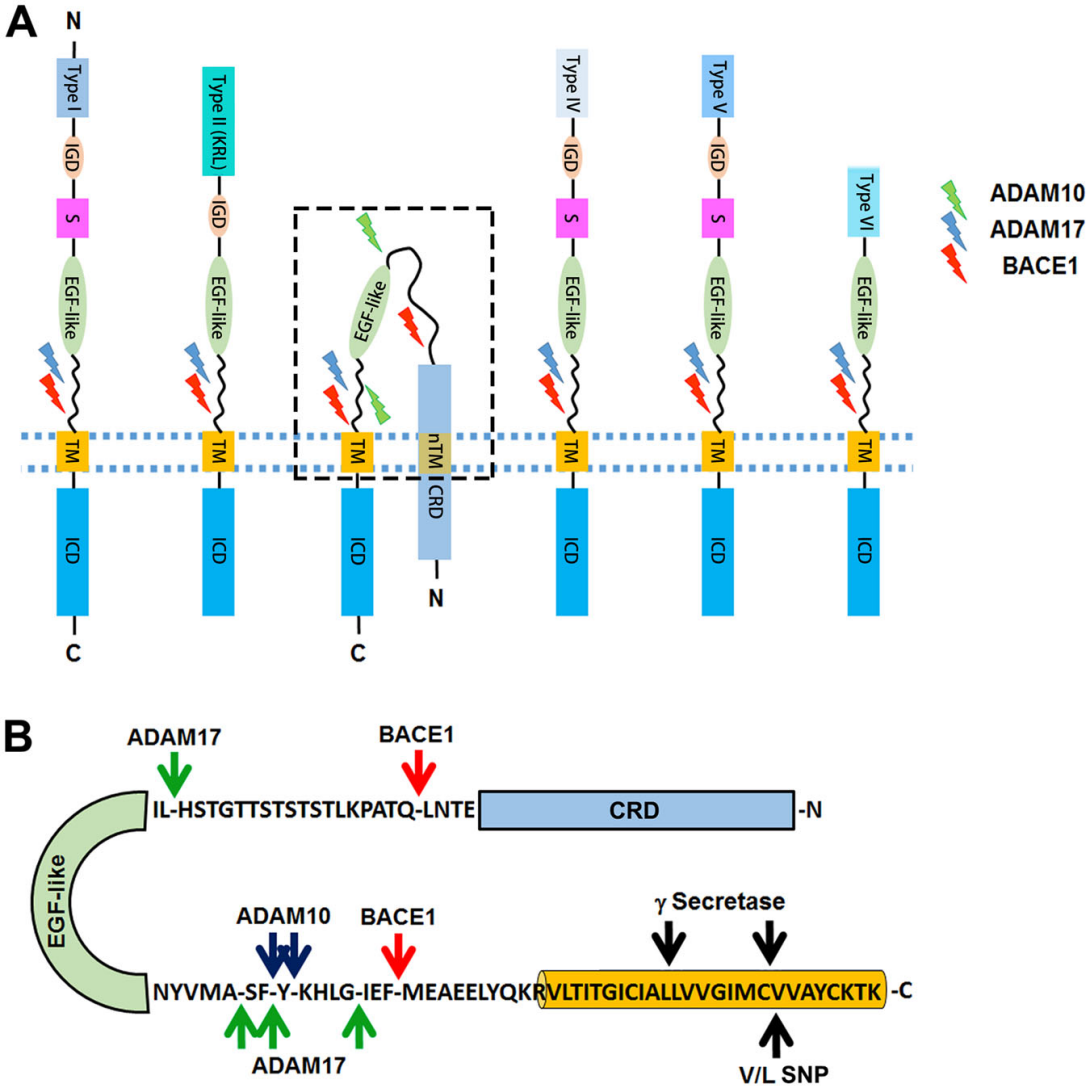
Functional domains and cleavage sites in NRG1. **a** Six classes of NRG1 structural variants are shown. Types I, II, IV, V, and VI are single TM proteins, whereas type III contains a cysteine-rich domain (CRD), which functions as a type II TM segment (nTM). Alternative splicing yields at least 15 unique N-termini containing a variety of functional domains including an EGF-like domain (EGF-like), immunoglobulin-like domain (IGD), and Kringle-like domain (KRL) as well as a ‘spacer’ region (S), tail segment, and linker region. The EGF-like domain is followed by three possible ‘tail’ segments encoded by exons α, β, and γ. Susceptibility to sheddase cleavage is specified by alternative splicing of four short exons in the linker region located upstream of the TM domain. Secreted isoforms lack the linker region due to in-frame stop codon in the γ exon. The KRL domain is present only in type II isoforms; the IGD mediates interactions with extracellular matrix heparin sulfate proteoglycans. The spacer contains multiple sites for N- and O-glycosylation. Alternative splicing results in three different ICDs referred to as a, b, and c. Flash symbols indicate the relative position of ADAM10 (blue), ADAM17 (green), and BACE1 (red) cleavage. **b** Segment of type III outlined by the box in panel **a**. Arrows point to ADAM10 (blue), ADAM17 (green), and BACE1(red) cleavage sites in the linker region or in the segment between the EGF-like and CRD domains. Cleavage sites for γ secretases (black arrow), and the position of the Sz-associated V/L SNP are shown in the TM domain.

Neurodevelopmental disorders including schizophrenia (Sz) and attention deficit hyperactivity disorder (ADHD) are chronic mental illnesses. For years, scientists have sought more effective treatment options for improving the cognitive outcomes in patients with Sz. The trophic factor Neuregulin 1 (NRG1, also called heuregulin1 or neu differentiation factor) activates signaling cascades, which regulate inhibitory neural processes important for executive functions, such as attention and working memory, which are impaired in Sz^1,2^. NRG1 is the best characterized of a four-member gene family (NRG1-NRG4) that all share an epidermal growth factor (EGF)-like domain with ErbB receptor binding activity (Fig. 1). With 33 exons, NRG1 isoform diversity is appreciable—most isoforms are synthesized as transmembrane (TM) pro-proteins that undergo proteolytic cleavage liberating a bioactive extracellular or ectodomain (ED) via a process called ‘ED shedding.’ Binding of the liberated NRG1 ED to the ErbB family of receptors stimulates signaling cascades involved in learning, memory, and other higher brain functions. Association studies link variants in the NRG1 gene with some of the cognitive deficits seen in Sz (reviewed in ref. ^3^); however, none meet genome wide significance for conferring an increased risk for the disorder^4–6^. The ErbB4 receptor subtype, in particular, plays a key role in the inhibitory functions of NRG1 in the CNS^7–11^. There is an extensive literature on the role of NRG1–ErbB4 signaling in early cortical development^12–15^ but see also ref. ^16^. In the following sections, we critically re-evaluate over two decades of research on the function of NRG1 in the mature central nervous system (CNS). We place particular emphasis on the complex role of NRG1–ErbB4 signaling in local circuit inhibitory neurons on cortical function and plasticity. We also examine the intrinsic and extrinsic factors regulating ED shedding, as this step holds the key to controlling NRG1 bioavailability. Finally, we develop a model for treating adults with Sz via gene therapies targeting NRG1/ErbB4 signaling in local circuit interneurons. Although much data support this model, we discuss the many challenges currently hindering translation of these types of therapeutic approaches.

## Critical periods of cortex development and neurodevelopmental disorders

**Fig. 2:**
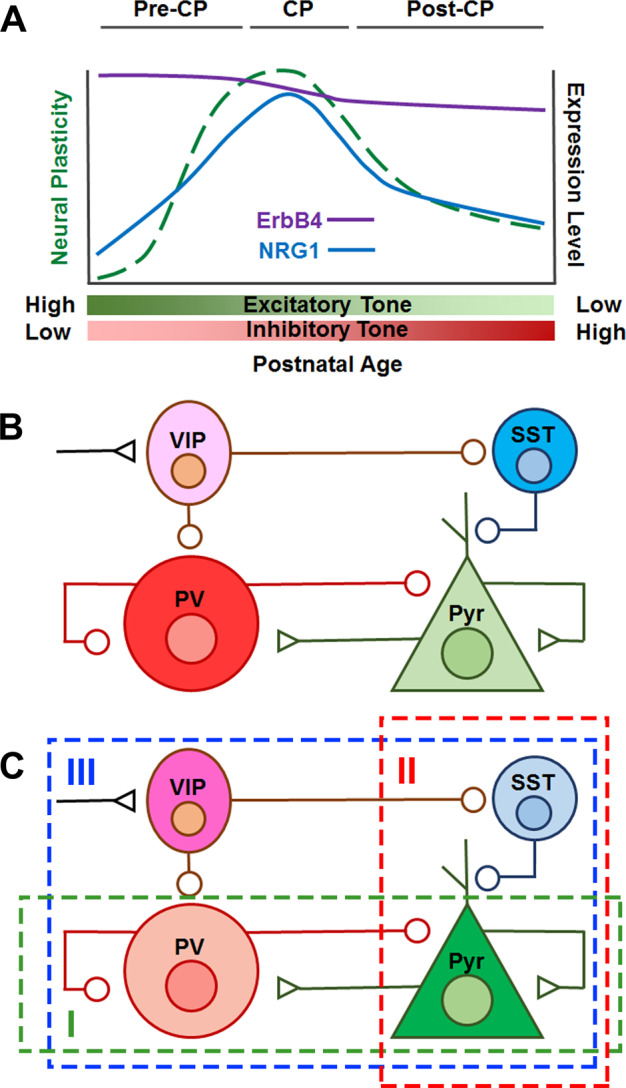
Critical period developmental of cortex. **a** Developmental changes in cortex during critical periods (CP). Neuroplasticity in cortex peaks during critical periods—local circuits undergo extensive remodeling as well as functional development. Expression level changes of ErbB4 (purple line) and NRG1 (blue line) across critical period development in mouse visual cortex^51^. The synaptic remodeling during critical period development results in an overall strengthening of inhibitory tone, and a reduction in neural plasticity. **b** Local circuit diagram showing connections made between fast-spiking PV+ basket cells, VIP+ interneurons, SST+ interneurons and excitatory pyramidal output neurons. Excitatory (triangle) and inhibitory (circle) inputs are shown. Fast-spiking PV+ basket cells inhibit pyramidal neurons through synapses made on the cell bodies, whereas SST+ neurons inhibit them via dendritic synapses. Both SST+ and PV+ neurons are under the control of VIP+ neurons^121^. Pyramidal cell firing is largely set by maturation of fast-spiking PV+ basket cells during critical periods^23^. **c** Three local circuit-based strategies that disinhibit pyramidal cells: (I) Decrease activity of fast-spiking PV+ basket cells (green box)^22^, (II) silence SST+ neurons (red box), or (III) activate VIP+ neurons (blue box)^29^.

Neurodevelopmental psychiatric disorders such as Sz typically emerge in late adolescence or early adulthood. This developmental epoch is considered to be a ‘critical period’ for maturation of prefrontal cortex (PFC), analogous to the postnatal timeframes defined for visual and other sensory systems^17,18^. Critical periods are also a time of heightened synaptic remodeling and integration of inhibitory interneurons into local neural circuits in cortex^19^(Fig. 2a). The best studied local circuit connects three main classes of interneurons (parvalbumin positive, PV+; somatostatin positive, SST+; and vasoactive intestinal peptide positive, VIP+) with excitatory pyramidal neurons, which are the principal output cells of cortex^20^ (Fig. 2b). During critical periods synaptic connections between interneurons and principal neurons are sculpted via mechanisms including pruning of dendritic spines on pyramidal cells and strengthening of excitatory synapses on PV+ and VIP+ classes of interneurons^11,18,21,22^. The coordinated activity of local circuit neurons plays a pivotal role in generating rhythmic patterns of neural activity in mature cortex. Fast-spiking PV+ basket cells, in particular, are crucial for the entrainment of gamma oscillations^23^. Oscillations in the gamma range (30–90 Hz) are associated with higher brain functions impaired in neurodevelopmental disorders such as working memory and attention^24^. Patients with Sz exhibit gamma oscillation abnormalities, which underscores the relevance of local circuit function as a therapeutic outcome^25–27^.

The extensive synaptic remodeling that occurs during critical period development decreases excitatory drive in cortex while enhancing inhibitory tone. As a result, neural circuits in cortex are less plastic after critical period closure (Fig. 2a). Additionally, other neural and genetic remodeling processes (e.g., myelination, perineuronal nets, and DNA methylation) come into play^18^. Along with increased inhibitory tone, these mechanisms are thought to constrain cortical plasticity, in effect limiting the learning and memory gains achievable with aging. Presumably, a persistent reduction in PFC plasticity in adulthood contributes to the failure of current therapies to rehabilitate the cognitive deficits associated with Sz^28^.

An exciting body of work in the visual system points to a set of local circuit neuron-based manipulations that can be performed after critical period closure to reactivate cortical plasticity (Fig. 2c). Initial findings revealed that manipulations that reduce excitatory input onto fast-spiking PV+ basket cells are effective^22^. Subsequent studies using transgenic and viral tools showed that either activating VIP+ interneurons or silencing SST+ interneurons represent other ways to disinhibit pyramidal cells and re-evoke plasticity^29^. Likewise, recent evidence indicates that manipulating NRG1–ErbB4 signaling in fast- spiking PV+ basket cells offers an additional option for overcoming reduced cortical excitability^30^. As discussed below, this and related findings from studies in VIP+ interneurons suggest that NRG1–ErbB4 signaling in interneurons could be poised to provide a tractable target for re-evoking cortical plasticity in PFC^11^.

## Crucial role of NRG1 in local circuit development and cortical plasticity

Several lines of evidence link NRG1 and ErbB4 with local circuit interneuron function. Foremost is the finding showing that a recombinant peptide corresponding to the EGF-like domain of NRG1 stimulates activity-dependent release of the inhibitory transmitter GABA^31^. This discovery set the stage for subsequent work demonstrating that the NRG1 EGF-like domain regulates not only gamma oscillations^1^, but also a host of related neural and behavioral phenomena including pyramidal cell excitability^2^, seizure activity^32–34^, synaptic plasticity^8,9,33,35^, and anxiety-like behavior^36^. The effects were shown in all cases to hinge on expression of ErbB4 receptors on PV+ neurons. The ability of the EGF-like domain peptide to suppress seizure activity and long-term synaptic potentiation (LTP) also implicates NRG1 in homeostatic mechanisms controlling excitatory and inhibitory (E/I) balance in neural circuits^9,34^.

Within cortex and hippocampus, ErbB4 receptors are predominantly expressed in interneurons, but also present in axonal projections of dopamine (DA) neurons as well as in oligodendrocytes^7,10,37^. As observed for PV+ basket cells, the vast majority of VIP+ interneurons in cortex prominently express ErbB4, whereas expression of the NRG1 receptor in cortical SST+ interneurons is sparse^10,11,37,38^ (Fig. 2c). Knockout of ErbB4 selectively in VIP+ interneurons abolishes the synchronized firing of fast-spiking PV+ and pyramidal neurons^11^. Thus, NRG1 activated signaling regulates the entrainment of oscillations in cortex via its actions on VIP+ interneurons in addition to its effects on fast-spiking PV+ basket cells. Studies with DA neuron-specific ErbB4 knockout mice indicate that dopaminergic expression of this NRG1 receptor is important for working memory; however, it is not yet clear if the mechanism involves modification of synchronized activity^39^.

Recent work indicates that NRG1 signaling also modulates the phase locking (neural synchrony) of local field potentials in ventral hippocampus and PFC^40^. This is relevant for neurodevelopmental disorders as ventral hippocampus-prefrontal synchrony in the theta and delta frequency ranges positively correlates with performance on the 5-choice serial reaction time test (5-CSRTT), which is a well-accepted task of attention^40,41^. Studies with ErbB4 ATP binding site mutant knockin mice indicate that ErbB4 receptors in ventral hippocampus and PFC play a role in performance on the 5-CSRTT as well as in ventral hippocampal-prefrontal synchrony^40^. These studies also showed that ErbB4 constitutive knockout mice (heart rescued) exhibit reduced hippocampal-prefrontal synchrony and deficits in attention^40^. Note, however, that another group reported no deficits in the performance of the ErbB4 constitutive knockout mice (heart rescued) on the 5-CSRTT relative to controls^42^. Other electrophysiological studies indicate, however, that interneuronal NRG1–ErbB4 signaling regulates neural synchrony in cortex^1,43,44^.

Although NRG1 enhances activity-dependent GABA transmission in cortex^31^, electrophysiological studies indicate that ErbB4 signaling in interneurons largely promotes maturation of glutamatergic inputs onto PV+ and VIP+ interneurons^11,30,45^. Consistent with this idea, ErbB4 receptors localize near excitatory inputs onto somatodendritic regions of interneurons^2,37,46,47^. Molecular studies suggest that NRG1–EGF-like domain binding stimulates ErbB4 receptor phosphorylation, which results in activation of a set of downstream signaling cascades (e.g., PI3 kinase/AKT, ERK, and perhaps others) (reviewed in ref. ^48^). Several studies indicate that ErbB4 signaling in interneurons stimulates incorporation of AMPA receptors into postsynaptic membranes, which is an established mechanism for potentiating excitatory transmission^49–51^. Two studies reported that ErbB4 signaling in interneurons preferentially regulates NMDA receptors. However, one study examined responses in a type I NRG1 transgenic mouse in which levels of ErbB4 in cortical interneurons are significantly downregulated^52^; the other study investigated the actions of NRG2^53^. Interaction of ErbB4 with PSD95, a postsynaptic scaffolding protein found at mature glutamatergic synapses, could also play a role in the mechanism as NRG1 signaling increases PSD95 levels near excitatory synapses on cortical interneurons in culture^50,54–56^. Altogether, these findings suggest that ErbB4 signaling regulates the molecular composition of excitatory synapses in interneurons via diverse mechanisms.

NRG1–ErbB4 signaling increases the firing of VIP+ and PV+ interneurons, in addition to promoting maturation of excitatory synapses on these cells. Although, the mechanism is not fully understood, the effect of ErbB4 signaling in PV+ interneurons is a reduction in both activity-dependent and spontaneous firing of pyramidal cells^2,45^. In contrast, ErbB4 signaling in VIP+ neurons regulates the synchronous firing of cortical pyramidal cells^11^. As discussed above, reduced pyramidal cell excitability is a key mechanism in critical period closure, as well as in the subsequent maintenance of reduced cortical plasticity (Fig. 2b). Intriguingly, a recent study on visual cortex showed that functional recovery of cortical plasticity in adults can be achieved by blocking NRG1 stimulation of ErbB4 receptors in PV+ neurons^30^. Hence, inhibiting ErbB4 activation in PV+ cells could be a viable alternative to the manipulations mentioned above for disinhibiting pyramidal cells after critical period closure^22,29^. Likewise, restoration of ErbB4 in VIP+ interneurons of the V1 area of visual cortex was found to rescue the cortical activity and visual performance deficits of mice with targeted deletion of ErbB4 in VIP+ cells^11^. These findings raise the intriguing possibility that selective targeting of NRG1 activation of ErbB4 in PV+ or VIP+ interneurons could also potentially improve PFC plasticity in adults.

## Developing NRG1-based gene therapies for increasing cortical plasticity in adults

Gene therapy of diseases of the CNS is an emerging technology offering the potential to manipulate the expression of specific molecules in select neural circuits by stereotactic delivery of recombinant viruses into various brain nuclei. Although invasive, recently completed Phase I and II clinical trials for Parkinson’s and Alzheimer’s diseases have demonstrated the safety of this therapeutic approach^57,58^. As discussed above, several lines of evidence from studies on primary sensory cortices support the potential efficacy of manipulating NRG1–ErbB4 signaling in local circuit interneurons for reactivating plasticity in mature cortex^11,29,30,51^. Moreover, these studies show that NRG1–ErbB4 signaling in PV+ and VIP+ local circuit interneurons regulates signal detection, noise filtering, information encoding, and rhythmic brain activity—all of which are critical for performance of executive functions like attention and working memory. In theory, therefore, these findings would seem to suggest that NRG1 and ErbB4 could be promising targets for gene therapy of neurodevelopmental disorders such as Sz. This idea is supported in principal by recent work showing that transgenic reinstatement of NRG1–ErbB4 signaling in adulthood can rescue some deficits in cortical GABA transmission caused by knockout of ErbB4 during early development^59^. To justify consideration as a therapeutic strategy, however, preclinical studies are needed to validate that manipulations of NRG–ErbB4 signaling in local circuit neurons can re-evoke cortical plasticity in higher order association area like PFC.

**Fig. 3:**
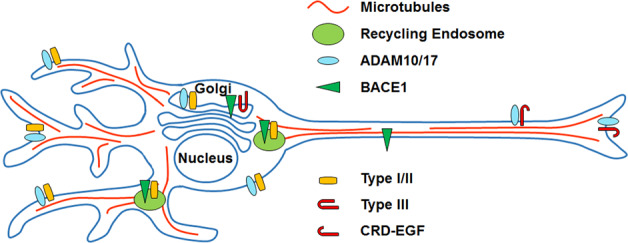
Polarized distribution of NRG1 isoforms in neurons. Differential subcellular localization of the three major NRG1 isoforms expressed in the CNS. Initial studies suggest that type I and II isoforms (Type I/II) are trafficked to the somatodendritic compartment^8,63,66^. In contrast, an N-terminal peptide fragment of type III containing the EGF-like and CRD domains (CRD-EGF) is sorted to axons following cleavage of the pro-protein (Type III) by BACE1 in the Golgi. BACE1, ADAM10, and ADAM17 have been shown to localize and/or to cleave substrates in both the somatodendritic and axonal compartments^122–127^.

In the following sections, we develop a circuit-based model for testing whether manipulating NRG1–ErbB4 signaling can reactivate plasticity of adult PFC. An advantage of circuit-based models is that they provide a framework for investigating alternate targets and signaling pathways^60^. The model developed here takes advantage of evidence linking local circuit interneuron with mechanisms that can reactivate excitability in cortex after critical period closure^22,29,30,61^. We propose two strategies for testing this model that focus on selectively increasing or decreasing ErbB4 signaling in PV+ or VIP+ interneurons, respectively. They explore the potential of targeting ErbB4 signaling local circuit interneurons as it is not yet known whether manipulating ErbB4 in other cell types (DA neurons or oligodendrocytes) can reactivate cortical plasticity after critical period closure. Neither strategy is intended to rescue any potential disease-related abnormalities in the NRG1 gene or anomalies its expression. The main driver of these strategies is the evidence (1) showing that cell-specific manipulation of ErbB4 receptors in either PV+ and VIP+ interneurons provides a means of tuning excitability of mature cortex^11,30,45,51^, and (2) indicating the differential cellular and subcellular distribution of NRG1 isoforms^62–66^ (Fig. 3). The model generates a number of testable hypotheses regarding the role of different NRG1 isoforms in maintaining the strength of glutamatergic synapse on PV+ and VIP+ interneurons in cortex.

Regional and cellular aspects of the model could be tested in either wild-type mice or mouse models of Sz^67^ via stereotactic injection of a recombinant adeno-associated virus (AAV) or lentivirus (LV) carrying a floxed cDNA or siRNA into PFC of adult transgenic mice in which CRE expression is conferred via a cell-specific promoter. AAVs and LVs are frequently the vectors of choice for gene therapy due to their stable long-term expression (up to years) and low risk of pathogenesis or cytotoxicity^68,69^. Use of transgenic CRE mice would enable testing of cell-type-specific predictions of the local circuit interneuron model. Regional specificity is critical because both the NRG1 and ErbB4 genes are expressed in a variety of cortical and subcortical brain regions^7,10,15,70^. Undesired side-effects resulting from global manipulation of a particular isoform in brain, even if cell-type-specific, could overshadow any potential benefits on prefrontal cortical plasticity. In support of this possibility, Sz-relevant behavioral and neuronal abnormalities were detected in transgenic mice that overexpress either the full length or just the bioactive ED of type I NRG1 in principal neurons throughout the brain^52,71–73^. Behavioral and neural deficits were also detected in transgenic mice with cell specific but brain-wide upregulation of other NRG1 isoforms (Table 1). For similar reasons, the ErbB2 monoclonal antibodies (Trastuzumab) that are used to treat Her2+ breast cancer, or the molecules (e.g., recombinant NRG1β and anti-ErbB4 antibodies) that are currently in development for heart disease and cancer might not be useful for neurodevelopmental disorders^74,75^. Moreover, systemic use of these agents could result in ‘off target’ complications due to, for example, the mitogenic effects of NRG1–ErbB signaling on the heart.

**Table 1:** Behavioral and neural abnormalities induced by NRG1 overexpression in pyramidal cells.

Isoform	Promoter	Cell type	Expression levels	Behavioral abnormalities	Synaptic alterations	Gamma oscillation alterations	Ref.
Type I	CamKII	PN	↑	H, WM, RM, SI, PPI	↓fEPSP ↓LTP ↑PPF ↓mEPSCs Freq ↓mIPSCs Amp	nd	^73^
Type I	Thy	PN	↑	H, WM with age	Normal fEPSP Normal LTP	↓Freq	^71^
Type I BACE1-cleaved ED	CamKII	PN	↑	H, WM, CFC, SI	nd	nd	^72^
Type III	CamKII	PN	↑	CFC, SI, PPI	nd	nd	^128^
Type III	Thy	PN	↑	nd	↓STP ↓LTP ↑sIPSCs Freq ↑mIPSCs Freq	nd	^76^

*PN* principal neuron, *H* hyperactive, *WM* working memory, *RM* reference memory, *SI* social interaction, *PPI* prepulse inhibition, *CFC* contextual fear conditioning, *fEPSP* field excitatory postsynaptic potential, *LTP* long-term potentiation, *PPF* paired-pulse facilitation, *mEPSCs* miniature excitatory postsynaptic currents, *mIPSCs* miniature inhibitory postsynaptic currents, *STP* short-term potentiation, *sIPSCs* spontaneous inhibitory postsynaptic currents, *Freq* frequency, *Amp* amplitude, *nd* not determined.

## Reduce NRG1 ED availability at excitatory synapses on PV+ basket cells

NRG1 signaling via ErbB4 maintains high levels of excitatory input onto fast-spiking PV+ basket cells, which is a key mechanism for dampening pyramidal cell activity in mature cortex^30,45^. One approach to increasing plasticity in adult PFC would therefore be to minimize NRG1 ED levels in the vicinity of excitatory synapses on PV+ basket cells. Several lines of evidence indicate that type III NRG1 isoforms, in particular, play an important role in maintaining excitatory input to fast-spiking PV+. For example, increased inhibition and reduced pyramidal neuron plasticity is detected in mice carrying a transgene that drives expression of type III cDNA in excitatory neurons^76^. In addition, reduced levels of type III NRG1 in excitatory neurons decreases glutamate transmission from these terminals^77^. Furthermore, the EGF-like domain of type III localizes to presynaptic terminals based on recombinant protein expression studies^64,66^, whereas type I and type II NRG1 isoforms localize to somatodendritic regions^63,66^ (Fig. 3).

Interestingly, BACE1 cleavage is critical for localization of the type III EGF-like domain in presynaptic terminals of excitatory neurons^63,64,66^. BACE1 is a transmembrane aspartyl protease that is most active in organelles with low lumenal pH such as the Golgi or early endosomes. Cleavage of type III pro-protein can be blocked by cell permeable inhibitors of BACE1, but not by membrane impermeant inhibitors of the enzyme suggesting that the proteolytic step could occur in the Golgi^62^. A protein fragment containing the EGF-like domain and upstream cysteine-rich domain (CRD) of type III is transported to axon terminals following ED cleavage in the stalk segment of the pro-protein^62,66^. Indeed, one study showed that inclusion of the EGF-like domain is necessary for presynaptic localization of the BACE1-cleaved type III fragment, highlighting the possibility that interaction with trans-synaptic ErbB4 receptors could play a role in the anchoring mechanism^66^. For single transmembrane NRG1 isoforms like type I and type II, sheddase cleavage in the linker liberates the EGF-like domain containing N-terminus (Fig. 1). Release of the bioactive EGF-like domain from cells can stimulate nearby ErbB receptors via either a paracrine or autocrine signaling mechanism. Extensive work, however, suggests that the EGF-like domain of type III isoforms remains membrane associated after sheddase cleavage due to the presence of the CRD which functions as a TM segment^78,79^ (Fig. 1). In some contexts, cell-attached configurations of the NRG1–EGF-like domain display bioactivity^80^. The recent discovery of additional BACE1 and ADAM17 cut sites between the CRD and EGF-like domains, however, indicates that type III isoforms might also be able to signal via a shedding-dependent mechanism^81^ (Fig. 1).

**Fig. 4:**
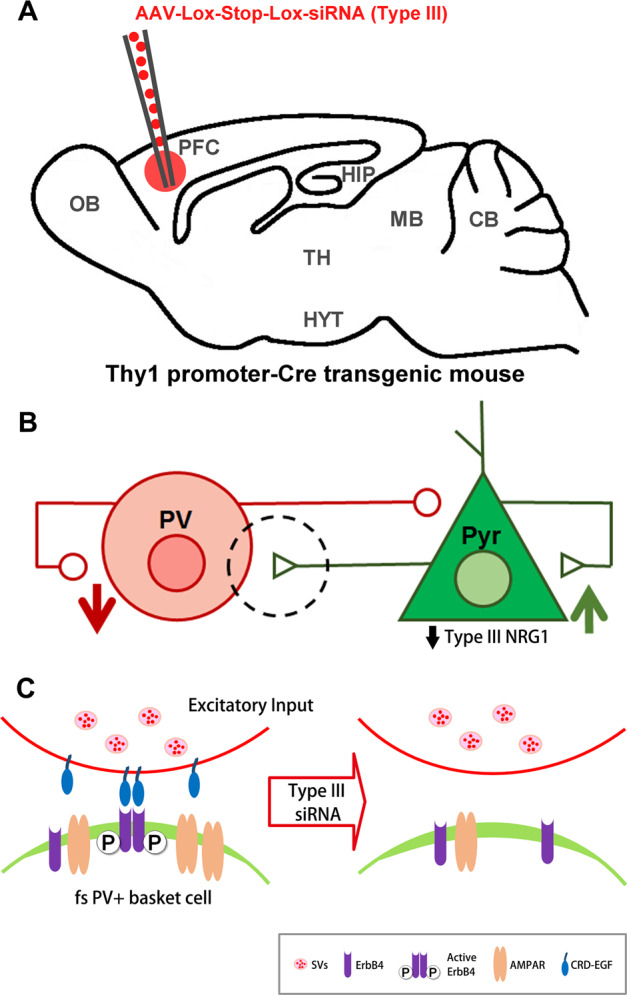
Fast-spiking PV+ basket cell-based strategy for reactivating cortical plasticity by lowering presynaptic type III NRG1 levels. **a** Stereotactic injection of AAV carrying a floxed type III NRG1 siRNA into PFC of Thy1-CRE transgenic mice. **b** Knockdown of type III in pyramidal cells is predicted to dampen the firing of fast-spiking PV+ basket cells and result in increased pyramidal cell excitability. **c** Enlargement of circled area outlined in **b**. siRNA KD should lower type III pro-protein levels in the Golgi and reduce subsequent accumulation of the type III CRD-EGF-like domain at excitatory inputs on PV+ neurons. This should limit presynaptic NRG1–EGF-like domain activation of postsynaptic ErbB4 receptors in PV+ neurons and subsequent insertion of synaptic AMPA receptors.

Silencing of type III in pyramidal neurons would be expected to preempt accumulation of the CRD-linked EGF-like domain at excitatory inputs onto fast-spiking PV+ basket cells. If the model shown in Fig. 4 is correct, this strategy should attenuate ErbB4 activation at these synapses, which should result in an increase in pyramidal cell activity and cortical plasticity. Type III silencing can be achieved by stereotactic injection of a recombinant AAV carrying a floxed type-III-specific short-interfering RNA (siRNA) into PFC of adult mice harboring a CRE transgene driven by a promoter expressed only in pyramidal cells in cortex such as Thy1^82^ (Fig. 4). There are a couple of features of type III transcripts that can be exploited in creating an isoform-selective reduction in type III via gene silencing strategies with siRNA. First, transcripts for type III derive from a unique promoter, whereas different promoters are used for transcribing other NRG1 isoforms^83^. Secondly, the exon encoding the CRD is found only in type III transcripts. Increased type III mRNA in PFC is associated with the HapICE risk haplotype for Sz found in the NRG1 gene^84–86^. If silencing type III in pyramidal cells in adult mice increases prefrontal plasticity and improves performance on tests of executive functions, then this strategy could potentially be a viable therapeutic approach to explore.

In addition to contacting local circuit interneurons via axon collaterals, PFC pyramidal cells project to a range of more distant cortical and subcortical targets that regulate cognitive and emotional processing, action selection, and reward behavior^87^. The consequences of type III KD on these behaviors are unknown, but as with any effects on local circuits, would presumably depend on ErbB4 expression in neurons targeted by these PFC projections. In this regard, there could be effects on regulation of fear and anxiety because ErbB4 expression is densest in the intercalated nucleus and medial region of the amygdala^10^. On the other hand, the effects of manipulating NRG1 could be attenuated as PFC projections appear to primarily target the anterior basal nucleus^88^. Another question is whether NRG1 type III KD could impact NRG3, which is also present in pyramidal axon terminals, in relatively close apposition to postsynaptic ErbB4 receptors^66,89^. Compared to NRG1, NRG3 is a much weaker (>10^4^-fold lower) agonist of ErbB4 tyrosine kinase activity^89^. Studies of KO mice indicate that NRG3 functions in excitatory terminals to promote synapse formation on interneurons, and to suppress glutamate release—however, neither function requires ErbB4^89,90^. Although none of the available data suggests that the functions of type III NRG1 and NRG3 are interdependent^66,89–93^, much remains to be learned about these different NRGs in local circuits. Below, we propose an alternate strategy for reactivating plasticity in adult PFC by manipulating NRG1–ErbB4 signaling in VIP+ interneurons.

## Increase NRG1 ED availability at excitatory synapses on VIP+ interneurons

**Fig. 5:**
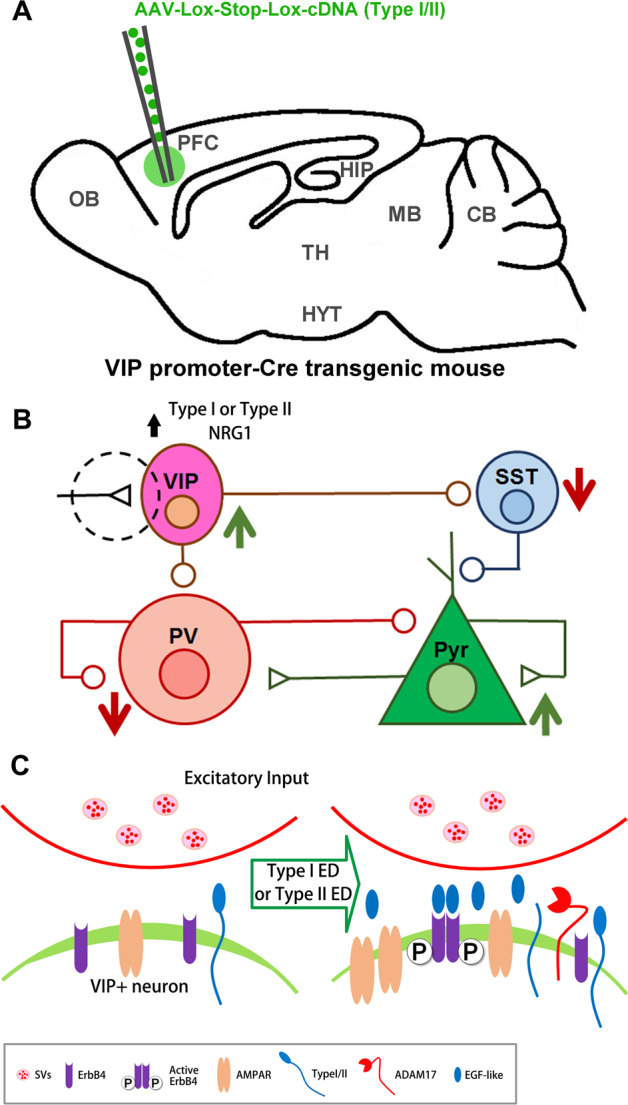
VIP+ interneuron-based strategy for reactivating cortical plasticity by increasing NRG1–EGF-like domain availability locally. **a** Stereotactic injection of AAV carrying floxed type I or type II NRG1 cDNA into PFC of VIP-CRE transgenic mice. **b** Increasing type I or II NRG1 availability in VIP+ neurons is predicted to disinhibit pyramidal cells by boosting ErbB4 receptor activity at excitatory synapses in these cells. **c** Enlargement of circled area outlined in **b**. Increased type I or type II ED release from VIP+ neurons should result in autocrine activation of ErbB4 receptors, and insertion of AMPA receptors. This should disinhibit pyramidal neurons by increasing firing of VIP+ neurons, which inhibit both SST+ interneurons and fast-spiking PV+ basket cells.

VIP+ interneurons enhance signal detection and noise filtering during arousal by modulating the basal excitability of pyramidal neurons—this translates into an improved ability of cortex to encode relevant new information^11,61^. In visual cortex at least, stimulating VIP+ neuronal activity is sufficient to disinhibit pyramidal cells, which is the central mechanism underlying reactivation of cortical plasticity^18,29^ (Fig. 2c). Furthermore, optogenetic studies indicate that selectively activating VIP+ interneurons dramatically improves performance on working memory tests^61^. Knockout of ErbB4 receptors selectively in VIP+ interneurons abolishes the synchronized firing of fast-spiking PV+ and pyramidal neurons in visual cortex^11^. Stimulating ErbB4 in VIP+ interneurons therefore potentially represents an alternative strategy for reactivating plasticity in PFC^11^ (Fig. 5).

VIP+ interneurons receive glutamatergic, cholinergic, serotonergic afferents from subcortical and other cortical areas^94–96^; however, ErbB4 receptors seem to only strengthen glutamatergic input onto these cells^11^. If the model shown in Fig. 5 is correct, pyramidal cell disinhibiton could be accomplished by microinjecting PFC of mice harboring a VIP+ promoter-driven CRE transgene with a recombinant AAV carrying a floxed cDNA encoding a sheddable isoform of NRG1, or the corresponding ED. Reasonable candidates include the type I and II isoforms as they are abundant in cortical GABAergic neurons^64^, and like ErbB4 receptors, these isoforms localize to neuronal somatodendritic compartments^63,65,66^ (Fig. 3). Type II could be a good first choice because glutamate stimulation promotes shedding of this isoform in transfected cortical neurons^63^. Additionally, the benefit of upregulating type I isoforms in VIP+ neurons is debatable. On the one hand, this isoform seems to undergo constitutive shedding, which could be advantageous for gene therapies designed to boost ErbB4 activity^78,79,97,98^. Nevertheless, concerns are raised by reports of upregulated type I NRG1 in cortex of patients with Sz^99–101^. However, the effect of upregulating type I exclusively in interneurons has yet to be examined.

Two studies have looked at mechanisms regulating NRG1 shedding from neurons^63,66^. One study of transfected neurons found that ADAM17 and protein kinase C (PKC) are required for neural activity-stimulated shedding of the bioactive ED^63^. More detailed mechanistic studies in non-neuronal cells show that PKC stimulates ED shedding (1) by increasing cell surface levels of ADAM17, and (2) by phosphorylating key residues in the intracellular domain (ICD) of NRG1^102^. These studies also showed that PKCδ-mediated phosphorylation of serine residue 286 in the NRG1 ICD is critical for ADAM17 cleavage^103,104^. ICD phosphorylation is proposed to initiate conformational changes in the NRG1 polypeptide that are relayed through the plasma membrane, and ultimately increase accessibility of the ADAM17 cleavage site in the ED^105,106^. Like NRG1, many proteins expressed in brain are substrates of ADAM17 or PKC (e.g., voltage gated Na+ channels, amyloid precursor protein and Notch/Delta), so upregulation of either enzyme would likely produce “off-target” side-effects^107–110^. On the other hand, knowledge of mechanisms promoting NRG1 shedding could be useful in the design of ED shedding-prone variants carrying mutations mimicking phosphorylation of key PKC sites in the ICD.

## Concluding remarks and future perspectives

Here we review data implicating NRG1–ErbB4 signaling in cortical plasticity and executive functions. We also explore the possibility of remediating cognitive and executive function deficits associated with Sz using gene therapy directed at NRG1–ErbB4 signaling in local circuits neurons in PFC. The therapeutic model that we develop takes advantage of cutting-edge information on local circuit mechanisms for reactivating excitability in cortex after critical period closure^22,29,30^. One advantage of this circuit-based approach is that it provides a framework for investigating alternate targets or signaling pathways. Another option would be to manipulate ErbB4 either in PV+ or VIP+ interneurons. Ideally, the biology underlying the illness will dictate which brain area should be targeted^111^. Justifications for targeting PFC include its well-established role in executive functions (e.g., cognitive flexibility, forward planning, and inhibition), which are significantly impaired in patients with Sz^112,113^. Further, stronger activation of PFC positively correlates with better functional outcomes for patients with Sz^114^. However, the primary neuroanatomical deficits of Sz are not well established. Indeed, meta-analyses suggest that in addition to PFC, improved outcomes correlate with increased resting-sate activity in several other brain regions (e.g., left hippocampus, superior temporal sulcus, and anterior and posterior cingulate cortex)^114–117^. It will be important to compare the relative efficacy of gene therapies injected into PFC versus these other fronto-limbic areas in improving executive functions. Postmortem neuropathological findings suggest that alterations in the cellular organization of cortex could be associated with Sz. Abnormalities reported range from a decreased density of inhibitory neurons to an increased number of proinflammatory cytokine-secreting microglia^118^. It is unclear whether these types of abnormalities would limit or accelerate possibilities for neural rehabilitation via the strategies proposed.

Even if the proposed preclinical studies validate the promise of these novel approaches, there are a number of issues that would still need to be addressed before gene therapy for Sz could be considered a near term possibility. One issue is that there is still much to be learned about the localization and function of the different members of the NRG gene family in the local circuits. Further so far only one study has looked at the impact of NRG1–ErbB4 signaling in VIP+ interneurons on cortical plasticity^11^. The manipulations that we propose should serve as a starting point for intervention. Better alternatives could very well arise as new discoveries are made that deepen our understanding of the NRG family in local circuits. Another concern is the absence of clinical trial data on the feasibility of stereotactic injection into brain of patients with Sz or other type of psychiatric illness. While a number of clinical studies have been carried out or are underway for a variety of neurological disorders^119^, use of these approaches for psychiatric disorders could potentially be thwarted by a number of obstacles due to the unique nature of these illnesses. For example, symptoms like paranoia and command auditory hallucinations are common in these disorders. Whether this patient population would be amenable to intracerebral surgical injection is unknown. Other issues that would need to be resolved relate to treatment duration and dosing given the chronic and heterogeneous nature of psychiatric illnesses. Phase I and II clinical trial data indicate cargo expression from intracerebrally injected recombinant AAV can persist for at least 2 years^57,58^. Although development of this therapeutic modality is still emerging, evidence from preclinical studies suggests both longer term expression and inducible expression are possible. For example, studies in primates show that expression from intracerebrally injected virus is maintained for up to 15 years without cytotoxicity or Lewy body formation^68^. Likewise, progress has been made on incorporating inducible genetic elements into AAV vectors, which would enable tighter control of cargo expression levels^120^.

In summary, despite over 50 years of research and development, the drugs available to treat Sz largely fail to remedy the cognitive and executive function deficits. Although there are still a number of open questions regarding feasibility as discussed above, gene therapy is a therapeutic alternative that should be explored given the intractability and chronic nature of these impairments.

## References

[1] Fisahn A (2009). Neuregulin-1 modulates hippocampal gamma oscillations: implications for schizophrenia. Cereb. Cortex.

[2] Wen L (2010). Neuregulin 1 regulates pyramidal neuron activity via ErbB4 in parvalbumin-positive interneurons. Proc. Natl Acad. Sci. USA.

[3] Mostaid MS (2016). Neuregulin-1 and schizophrenia in the genome-wide association study era. Neurosci. Biobehav. Rev..

[4] Farrell MS (2015). Evaluating historical candidate genes for schizophrenia. Mol. Psychiatry.

[5] Schizophrenia Working Group of the Psychiatric Genomics Consortium. (2014). Biological insights from 108 schizophrenia-associated genetic loci. Nature.

[6] Pardiñas AF (2018). Common schizophrenia alleles are enriched in mutation-intolerant genes and in regions under strong background selection. Nat. Genet..

[7] Neddens J, Buonanno A (2011). Expression of the neuregulin receptor ErbB4 in the brain of the rhesus monkey (Macaca mulatta). PloS ONE.

[8] Shamir A (2012). The importance of the NRG-1/ErbB4 pathway for synaptic plasticity and behaviors associated with psychiatric disorders. J. Neurosci. J. Soc. Neurosci..

[9] Chen Y-J (2010). ErbB4 in parvalbumin-positive interneurons is critical for neuregulin 1 regulation of long-term potentiation. Proc. Natl Acad. Sci. USA.

[10] Bean JC (2014). Genetic labeling reveals novel cellular targets of schizophrenia susceptibility gene: distribution of GABA and non-GABA ErbB4-positive cells in adult mouse brain. J. Neurosci. J. Soc. Neurosci..

[11] Batista-Brito R (2017). Developmental dysfunction of VIP interneurons impairs cortical circuits. Neuron.

[12] López-Bendito G (2006). Tangential neuronal migration controls axon guidance: a role for neuregulin-1 in thalamocortical axon navigation. Cell.

[13] Flames N (2004). Short- and long-range attraction of cortical GABAergic interneurons by neuregulin-1. Neuron.

[14] Hanashima C (2006). Building bridges to the cortex. Cell.

[15] Yau H-J (2003). Neural development of the neuregulin receptor ErbB4 in the cerebral cortex and the hippocampus: preferential expression by interneurons tangentially migrating from the ganglionic eminences. Cereb. Cortex N. Y. N. 1991.

[16] Barros CS (2009). Impaired maturation of dendritic spines without disorganization of cortical cell layers in mice lacking NRG1/ErbB signaling in the central nervous system. Proc. Natl Acad. Sci. USA.

[17] Selemon LD, Zecevic N (2015). Schizophrenia: a tale of two critical periods for prefrontal cortical development. Transl. Psychiatry.

[18] Werker JF, Hensch TK (2015). Critical periods in speech perception: new directions. Annu. Rev. Psychol..

[19] Trachtenberg JT (2015). Competition, inhibition, and critical periods of cortical plasticity. Curr. Opin. Neurobiol..

[20] Cardin JA (2018). Inhibitory interneurons regulate temporal precision and correlations in cortical circuits. Trends Neurosci..

[21] Bourgeois JP (1994). Synaptogenesis in the prefrontal cortex of rhesus monkeys. Cereb. Cortex.

[22] Kuhlman SJ (2013). A disinhibitory microcircuit initiates critical-period plasticity in the visual cortex. Nature.

[23] Cardin JA (2009). Driving fast-spiking cells induces gamma rhythm and controls sensory responses. Nature.

[24] Buzsáki G, Wang X-J (2012). Mechanisms of gamma oscillations. Annu. Rev. Neurosci..

[25] Wang X-J (2010). Neurophysiological and computational principles of cortical rhythms in cognition. Physiol. Rev..

[26] Gonzalez-Burgos G (2015). Alterations in cortical network oscillations and parvalbumin neurons in schizophrenia. Biol. Psychiatry.

[27] Lewis DA (2012). Cortical parvalbumin interneurons and cognitive dysfunction in schizophrenia. Trends Neurosci..

[28] Hill SK (2010). Effect of second-generation antipsychotics on cognition: current issues and future challenges. Expert Rev. Neurother..

[29] Fu Y (2015). A cortical disinhibitory circuit for enhancing adult plasticity. eLife.

[30] Gu Y (2016). Neuregulin-dependent regulation of fast-spiking interneuron excitability controls the timing of the critical period. J. Neurosci. J. Soc. Neurosci..

[31] Woo R-S (2007). Neuregulin-1 enhances depolarization-induced GABA release. Neuron.

[32] Eilam R (1998). Activity-dependent regulation of Neu differentiation factor/neuregulin expression in rat brain. Proc. Natl Acad. Sci. USA.

[33] Iyengar SS, Mott DD (2008). Neuregulin blocks synaptic strengthening after epileptiform activity in the rat hippocampus. Brain Res..

[34] Tan G-H (2012). Neuregulin 1 represses limbic epileptogenesis through ErbB4 in parvalbumin-expressing interneurons. Nat. Neurosci..

[35] Kwon O-B (2005). Neuregulin-1 reverses long-term potentiation at CA1 hippocampal synapses. J. Neurosci..

[36] Bi L-L (2015). Amygdala NRG1-ErbB4 is critical for the modulation of anxiety-like behaviors. Neuropsychopharmacol. Publ. Am. Coll. Neuropsychopharmacol..

[37] Neddens J (2011). Conserved interneuron-specific ErbB4 expression in frontal cortex of rodents, monkeys, and humans: implications for schizophrenia. Biol. Psychiatry.

[38] Ahrens S (2015). ErbB4 regulation of a thalamic reticular nucleus circuit for sensory selection. Nat. Neurosci..

[39] Skirzewski M (2018). ErbB4 signaling in dopaminergic axonal projections increases extracellular dopamine levels and regulates spatial/working memory behaviors. Mol. Psychiatry.

[40] Tan Z (2018). Dynamic ErbB4 activity in hippocampal-prefrontal synchrony and top-down attention in rodents. Neuron.

[41] Robbins TW (2002). The 5-choice serial reaction time task: behavioural pharmacology and functional neurochemistry 1 288. Psychopharmacol. Berl..

[42] Marchisella E (2018). Constitutive loss and acute pharmacological manipulation of ErbB4 signaling do not affect attention and inhibitory control in mice. Genes Brain Behav..

[43] Hou X-J (2014). Neuregulin 1/ErbB4 enhances synchronized oscillations of prefrontal cortex neurons via inhibitory synapses. Neuroscience.

[44] Kawata M (2017). Ablation of neuropsin-neuregulin 1 signaling imbalances ErbB4 inhibitory networks and disrupts hippocampal gamma oscillation. Transl. Psychiatry.

[45] Yang J-M (2013). Development of GABA circuitry of fast-spiking basket interneurons in the medial prefrontal cortex of erbb4-mutant mice. J. Neurosci. J. Soc. Neurosci..

[46] Vullhorst D (2009). Selective expression of ErbB4 in interneurons, but not pyramidal cells, of the rodent hippocampus. J. Neurosci. J. Soc. Neurosci..

[47] Krivosheya D (2008). ErbB4-neuregulin signaling modulates synapse development and dendritic arborization through distinct mechanisms. J. Biol. Chem..

[48] Mei L, Nave K-A (2014). Neuregulin-ERBB signaling in the nervous system and neuropsychiatric diseases. Neuron.

[49] Del Pino I (2013). Erbb4 deletion from fast-spiking interneurons causes schizophrenia-like phenotypes. Neuron.

[50] Li B (2007). The neuregulin-1 receptor erbB4 controls glutamatergic synapse maturation and plasticity. Neuron.

[51] Sun Y (2016). Neuregulin-1/ErbB4 signaling regulates visual cortical plasticity. Neuron.

[52] Kotzadimitriou, D. et al. Neuregulin 1 type I overexpression is associated with reduced NMDA receptor-mediated synaptic signaling in hippocampal interneurons expressing PV or CCK. *eNeuro* **5**, ENEURO.0418-17.2018 (2018). 10.1523/ENEURO.0418-17.2018 PMC5938717 29740596

[53] Vullhorst D (2015). A negative feedback loop controls NMDA receptor function in cortical interneurons via neuregulin 2/ErbB4 signalling. Nat. Commun..

[54] Huang YZ (2000). Regulation of neuregulin signaling by PSD-95 interacting with ErbB4 at CNS synapses. Neuron.

[55] Garcia RA (2000). The neuregulin receptor ErbB-4 interacts with PDZ-containing proteins at neuronal synapses. Proc. Natl Acad. Sci. USA.

[56] Ting AK (2011). Neuregulin 1 promotes excitatory synapse development and function in GABAergic interneurons. J. Neurosci. J. Soc. Neurosci..

[57] Hitti, F. L. et al. (2019) Human gene therapy approaches for the treatment of Parkinson’s disease: an overview of current and completed clinical trials. *Parkinsonism Relat. Disord*. 10.1016/j.parkreldis.2019.07.018 (2019). 10.1016/j.parkreldis.2019.07.018 31324556

[58] Rafii MS (2018). Adeno-associated viral vector (serotype 2)-nerve growth factor for patients with Alzheimer disease: a randomized clinical trial. JAMA Neurol..

[59] Wang H (2018). Genetic recovery of ErbB4 in adulthood partially restores brain functions in null mice. Proc. Natl Acad. Sci. USA.

[60] Gordon JA (2016). On being a circuit psychiatrist. Nat. Neurosci..

[61] Kamigaki T, Dan Y (2017). Delay activity of specific prefrontal interneuron subtypes modulates memory-guided behavior. Nat. Neurosci..

[62] Ben Halima S (2016). Specific inhibition of β-secretase processing of the Alzheimer disease amyloid precursor protein. Cell Rep..

[63] Iwakura Y (2017). Glutamate-dependent ectodomain shedding of neuregulin-1 type II precursors in rat forebrain neurons. PloS ONE.

[64] Liu X (2011). Specific regulation of NRG1 isoform expression by neuronal activity. J. Neurosci. J. Soc. Neurosci..

[65] Shamir A, Buonanno A (2010). Molecular and cellular characterization of Neuregulin-1 type IV isoforms. J. Neurochem..

[66] Vullhorst D (2017). Structural similarities between neuregulin 1-3 isoforms determine their subcellular distribution and signaling mode in central neurons. J. Neurosci. J. Soc. Neurosci..

[67] Hamm JP (2017). Altered cortical ensembles in mouse models of schizophrenia. Neuron.

[68] Sehara Y (2017). Persistent expression of dopamine-synthesizing enzymes 15 years after gene transfer in a primate model of Parkinson’s disease. Hum. Gene Ther. Clin. Dev..

[69] Marquez Loza LI (2019). Lentiviral vectors for the treatment and prevention of cystic fibrosis lung disease. Genes.

[70] Law AJ (2004). Neuregulin-1 (NRG-1) mRNA and protein in the adult human brain. Neuroscience.

[71] Deakin, I. H. et al. Transgenic overexpression of the type I isoform of neuregulin 1 afects working Memory and hippocampal oscillations but not long-term potentiation. *Cereb. Cortex*10.1093/cercor/bhr223 (2011). 10.1093/cercor/bhr223 PMC3377963 21878485

[72] Luo X (2013). Reversible overexpression of Bace1-cleaved neuregulin-1 N-terminal fragment induces schizophrenia-like phenotypes in mice. Biol. Psychiatry.

[73] Yin D-M (2013). Reversal of behavioral deficits and synaptic dysfunction in mice overexpressing neuregulin 1. Neuron.

[74] Galindo CL (2014). Neuregulin as a heart failure therapy and mediator of reverse remodeling. Curr. Heart Fail. Rep..

[75] Okazaki S (2016). Development of an ErbB4 monoclonal antibody that blocks neuregulin-1-induced ErbB4 activation in cancer cells. Biochem. Biophys. Res. Commun..

[76] Agarwal A (2014). Dysregulated expression of neuregulin-1 by cortical pyramidal neurons disrupts synaptic plasticity. Cell Rep..

[77] Zhong, C. et al. Axonal type III Nrg1 controls glutamate synapse formation and GluA2 trafficking in hippocampal-accumbens connections. *eNeuro* **4**, ENEURO.0232-16.2017 (2017). 10.1523/ENEURO.0232-16.2017 PMC5329619 28275713

[78] Burgess TL (1995). Biosynthetic processing of neu differentiation factor. glycosylation trafficking, and regulated cleavage from the cell surface. J. Biol. Chem..

[79] Wang JY (2001). The N-terminal region of neuregulin isoforms determines the accumulation of cell surface and released neuregulin ectodomain. J. Biol. Chem..

[80] Montero JC (2011). Transautocrine signaling by membrane neuregulins requires cell surface targeting, which is controlled by multiple domains. J. Biol. Chem..

[81] Fleck D (2013). Dual cleavage of neuregulin 1 type III by BACE1 and ADAM17 liberates its EGF-like domain and allows paracrine signaling. J. Neurosci..

[82] Heimer-McGinn V, Young P (2011). Efficient inducible Pan-neuronal cre-mediated recombination in SLICK-H transgenic mice. Genesis.

[83] Chen Y-JJ (2008). Type III neuregulin-1 is required for normal sensorimotor gating, memory-related behaviors, and corticostriatal circuit components. J. Neurosci. J. Soc. Neurosci..

[84] Stefansson H (2002). Neuregulin 1 and susceptibility to schizophrenia. Am. J. Hum. Genet..

[85] Weickert CS (2012). Schizophrenia-associated HapICE haplotype is associated with increased NRG1 type III expression and high nucleotide diversity. Transl. Psychiatry.

[86] Stefansson H (2004). Neuregulin 1 and schizophrenia. Ann. Med..

[87] Haber, S. N. et al. Circuits, networks, and neuropsychiatric disease: transitioning from anatomy to imaging. *Biol. Psychiatry*10.1016/j.biopsych.2019.10.024 (2019). 10.1016/j.biopsych.2019.10.024 31870495

[88] Arruda-Carvalho M, Clem RL (2015). Prefrontal-amygdala fear networks come into focus. Front. Syst. Neurosci.

[89] Müller, T. et al. Neuregulin 3 promotes excitatory synapse formation on hippocampal interneurons. *EMBO J.* **37**, e98858 (2018). 10.15252/embj.201798858 PMC6120667 30049711

[90] Wang Y-N (2018). Controlling of glutamate release by neuregulin3 via inhibiting the assembly of the SNARE complex. Proc. Natl Acad. Sci. USA.

[91] Grieco SF (2020). Neuregulin and ErbB expression is regulated by development and sensory experience in mouse visual cortex. J. Comp. Neurol..

[92] Loos M (2014). Neuregulin-3 in the mouse medial prefrontal cortex regulates impulsive action. Biol. Psychiatry.

[93] Paterson C, Law AJ (2014). Transient overexposure of neuregulin 3 during early postnatal development impacts selective behaviors in adulthood. PloS ONE.

[94] Fu Y (2014). A cortical circuit for gain control by behavioral state. Cell.

[95] Lee S (2013). A disinhibitory circuit mediates motor integration in the somatosensory cortex. Nat. Neurosci..

[96] Wall NR (2016). Brain-wide maps of synaptic input to cortical interneurons. J. Neurosci. J. Soc. Neurosci..

[97] Zhang Z (2006). Neuregulin isoforms in dorsal root ganglion neurons: effects of the cytoplasmic domain on localization and membrane shedding of Nrg-1 type I. J. Neurosci. Res..

[98] Loeb JA (1998). The neuregulin precursor proARIA is processed to ARIA after expression on the cell surface by a protein kinase C-enhanced mechanism. Mol. Cell. Neurosci..

[99] Hashimoto R (2003). Expression analysis of neuregulin-1 in the dorsolateral prefrontal cortex in schizophrenia. Mol. Psychiatry.

[100] Law AJ (2006). Neuregulin 1 transcripts are differentially expressed in schizophrenia and regulated by 5’ SNPs associated with the disease. Proc. Natl Acad. Sci. USA.

[101] Chong VZ (2008). Elevated neuregulin-1 and ErbB4 protein in the prefrontal cortex of schizophrenic patients. Schizophr. Res..

[102] Lorenzen I (2016). Control of ADAM17 activity by regulation of its cellular localisation. Sci. Rep..

[103] Dang M (2011). Epidermal growth factor (EGF) ligand release by substrate-specific a disintegrin and metalloproteases (ADAMs) involves different protein kinase C (PKC) isoenzymes depending on the stimulus. J. Biol. Chem..

[104] Dang M (2013). Regulated ADAM17-dependent EGF family ligand release by substrate-selecting signaling pathways. Proc. Natl Acad. Sci. USA.

[105] Hartmann M (2015). Inside-out regulation of ectodomain cleavage of cluster-of-differentiation-44 (CD44) and of neuregulin-1 requires substrate dimerization. J. Biol. Chem..

[106] Parra LM (2015). Distinct intracellular domain substrate modifications selectively regulate ectodomain cleavage of NRG1 or CD44. Mol. Cell. Biol..

[107] Kuhn P-H (2016). Systematic substrate identification indicates a central role for the metalloprotease ADAM10 in axon targeting and synapse function. eLife.

[108] Kim DY (2007). BACE1 regulates voltage-gated sodium channels and neuronal activity. Nat. Cell Biol..

[109] Kuhn P-H (2012). Secretome protein enrichment identifies physiological BACE1 protease substrates in neurons. EMBO J..

[110] Prox J (2013). Postnatal disruption of the disintegrin/metalloproteinase ADAM10 in brain causes epileptic seizures, learning deficits, altered spine morphology, and defective synaptic functions. J. Neurosci. J. Soc. Neurosci..

[111] Gelfand Y, Kaplitt MG (2013). Gene therapy for psychiatric disorders. World Neurosurg..

[112] Yuan P, Raz N (2014). Prefrontal cortex and executive functions in healthy adults: a meta-analysis of structural neuroimaging studies. Neurosci. Biobehav. Rev..

[113] Thai ML (2019). A meta-analysis of executive dysfunction in patients with schizophrenia: different degree of impairment in the ecological subdomains of the Behavioural Assessment of the Dysexecutive Syndrome. Psychiatry Res..

[114] Wojtalik JA (2017). A systematic and meta-analytic review of neural correlates of functional outcome in schizophrenia. Schizophr. Bull..

[115] Kühn S, Gallinat J (2013). Resting-state brain activity in schizophrenia and major depression: a quantitative meta-analysis. Schizophr. Bull..

[116] Dong D (2018). Dysfunction of large-scale brain networks in schizophrenia: a meta-analysis of resting-state functional connectivity. Schizophr. Bull..

[117] Crossley NA (2016). Altered hub functioning and compensatory activations in the connectome: a meta-analysis of functional neuroimaging studies in schizophrenia. Schizophr. Bull..

[118] Bakhshi K, Chance SA (2015). The neuropathology of schizophrenia: a selective review of past studies and emerging themes in brain structure and cytoarchitecture. Neuroscience.

[119] Haggerty DL (2020). Adeno-associated viral vectors in neuroscience research. Mol. Ther. Methods Clin. Dev..

[120] O’Callaghan J (2017). Therapeutic potential of AAV-mediated MMP-3 secretion from corneal endothelium in treating glaucoma. Hum. Mol. Genet..

[121] Williams LE, Holtmaat A (2019). Higher-order thalamocortical inputs gate synaptic long-term potentiation via disinhibition. Neuron.

[122] Buggia-Prévot V (2014). Axonal BACE1 dynamics and targeting in hippocampal neurons: a role for Rab11 GTPase. Mol. Neurodegener..

[123] Cho RW (2008). mGluR1/5-dependent long-term depression requires the regulated ectodomain cleavage of neuronal pentraxin NPR by TACE. Neuron.

[124] Das U (2013). Activity-induced convergence of APP and BACE-1 in acidic microdomains via an endocytosis-dependent pathway. Neuron.

[125] Lundgren JL (2015). ADAM10 and BACE1 are localized to synaptic vesicles. J. Neurochem..

[126] Saraceno C (2014). SAP97-mediated ADAM10 trafficking from Golgi outposts depends on PKC phosphorylation. Cell Death Dis..

[127] Zunke F, Rose-John S (2017). The shedding protease ADAM17: physiology and pathophysiology. Biochim. Biophys. Acta Mol. Cell Res..

[128] Olaya JC (2018). Overexpression of neuregulin 1 type III confers hippocampal mRNA alterations and schizophrenia-like behaviors in mice. Schizophr. Bull..

